# Similar CD4/CD8 Ratio Recovery After Initiation of Dolutegravir Plus Lamivudine Versus Dolutegravir or Bictegravir-Based Three-Drug Regimens in Naive Adults With HIV

**DOI:** 10.3389/fimmu.2022.873408

**Published:** 2022-03-31

**Authors:** Javier Martínez-Sanz, Raquel Ron, Elena Moreno, Matilde Sánchez-Conde, Alfonso Muriel, Luis Fernando López Cortés, José Ramón Blanco, Juan Antonio Pineda, Álvaro Mena, Sonia Calzado Isbert, Santiago Moreno, Sergio Serrano-Villar

**Affiliations:** ^1^ Department of Infectious Diseases, Hospital Universitario Ramón y Cajal, Instituto Ramón y Cajal de Investigación Sanitaria (IRYCIS), Madrid, Spain; ^2^ CIBER de Enfermedades Infecciosas (CIBERINFEC), Instituto de Salud Carlos III, Madrid, Spain; ^3^ Biostatistics Unit, Hospital Universitario Ramón y Cajal, Instituto Ramón y Cajal de Investigación Sanitaria (IRYCIS), Madrid, Spain; ^4^ CIBER Epidemiología y Salud Pública (CIBERESP), Instituto de Salud Carlos III, Madrid, Spain; ^5^ University of Alcalá, Madrid, Spain; ^6^ Clinical Unit of Infectious Diseases, Microbiology and Preventive Medicine, Hospital Universitario Virgen del Rocío, Sevilla, Spain; ^7^ Hospital San Pedro, Centro de Investigación Biomédica de la Rioja (CIBIR), Logroño, Spain; ^8^ Department of Infectious Diseases and Clinical Microbiology, Hospital Universitario Nuestra Señora de Valme, Sevilla, Spain; ^9^ Infectious Diseases Unit, Internal Medicine Service, Complejo Hospitalario Universitario de A Coruña (CHUAC), A Coruña, Spain; ^10^ Department of Infectious Diseases, Corporació Sanitària Parc Taulí, Sabadell, Spain

**Keywords:** HIV, antiretroviral therapy, dual therapy, CD4/CD8 ratio, integrase inhibitors

## Abstract

**Background:**

The initiation of antiretroviral treatment based on a 2-drug regimen (2DR) with dolutegravir plus lamivudine has demonstrated non-inferior efficacy than dolutegravir-based three-drug regimens (3DR). We aimed to assess whether the treatment initiation with this 2DR has a different impact on the CD4/CD8 ratio recovery than INSTI-based 3DR.

**Methods:**

We emulated a target trial using observational data from the Spanish HIV Research Network cohort (CoRIS). The outcomes of interest were the normalization of the CD4/CD8 ratio at 48 weeks using three different cutoffs: 0.5, 1.0, and 1.5. We matched each participant who started 2DR with up to four participants who received 3DR. Subsequently, we fitted generalized estimating equation (GEE) models and used the Kaplan–Meier method for survival curves.

**Results:**

We included 485, 805, and 924 participants for cutoffs of 0.5, 1.0, and 1.5, respectively. At 48 weeks, 45% of participants achieved a CD4/CD8 ratio >0.5, 15% achieved a ratio >1.0, and 6% achieved a ratio >1.5. GEE models yielded a similar risk of reaching a CD4/CD8 ratio >0.5 (OR 1.00, 95% CI 0.67 - 1.50), CD4/CD8 >1.0 (OR 1.03, 95% CI 0.68 - 1.58), and CD4/CD8 >1.5 (OR 0.86, 95% CI 0.48 - 1.54) between both treatment strategies. There were no differences between 2DR and 3DR in the incidence ratio of CD4/CD8 ratio normalization at 0.5, 1.0 and 1.5 cut-offs.

**Conclusions:**

In this large cohort study in people with HIV, ART initiation with dolutegravir plus lamivudine vs. dolutegravir or bictegravir-based triple antiretroviral therapy showed no difference in the rates of CD4/CD8 normalization at 48 weeks.

## Background

The initiation of antiretroviral treatment (ART) based on a 2-drug regimen (2DR) with dolutegravir (DTG) plus lamivudine (3TC) has demonstrated non-inferior efficacy and a similar tolerability profile than the 3DR based on the combination of DTG plus tenofovir disoproxil fumarate (TDF) and emtricitabine (FTC) (1). However, further information is warranted about possible differentiating factors.

Analyzing the effect of therapeutic strategies on the CD4/CD8 ratio is clinically relevant. The persistence of high levels of circulating CD8+ T-cells or a low CD4/CD8 ratio translates into immunosenescence and heightened immune activation (2) and has been associated with an excess risk of non-AIDS comorbidities and higher all-cause mortality in people living with HIV (PLHIV) receiving ART (2–4). On the other hand, patients with a normal CD4/CD8 ratio (>1) show levels of immune activation and immunosenescence similar to those observed in the non-HIV-infected population. For these reasons, the CD4/CD8 ratio has been established as a marker of immune recovery in people receiving ART and, therefore, as an important prognostic marker. Some (5), but not all (6), clinical guidelines already recommend monitoring the CD4/CD8 ratio as a better predictor of adverse outcomes than the CD4+ T-cell count.

Accumulating evidence suggests that integrase strand-transfer inhibitors (INSTI)-based initial ART results in greater CD4/CD8 ratio recovery compared to non-nucleoside reverse transcriptase (NNRTI) or protease inhibitors (PI)-based ART, with a greater effect during the first year of therapy (3, 7). However, it is unknown whether the treatment initiation with INSTI-based 2DR has a different impact on the CD4/CD8 ratio recovery compared to INSTI-based 3DR. Some studies have been performed in the switch setting and usually group different drug combinations within the 2DR, many based on protease inhibitors (8–11). Hence, we show here the effects of initial INSTI-based 2DR versus 3DR in a large prospective cohort of ART-naïve PLHIV.

## Methods

### Study Design, Participants, and Setting

We designed this observational study to emulate a target trial of the causal effect of two different INSTI-based ART strategies on CD4/CD8 ratio normalization. The three outcomes of interest were the normalization of the CD4/CD8 ratio at 48 weeks using three different cutoffs: 0.5, 1.0, and 1.5. The rationale for the use of these cutoff points was described elsewhere (7). We collected data from 17,683 treatment-naïve adult patients recruited from 45 Spanish hospitals and prospectively included in the Cohort of the Spanish HIV Research Network (CoRIS). We included all subjects who started 2DR with DTG plus 3TC or 3DR with bictegravir (BIC) plus tenofovir alafenamide (TAF) and FTC, or DTG plus two nucleoside reverse transcriptase inhibitors (3TC and abacavir, TDF and FTC, or TAF and FTC). To ensure exchangeability, we included participants from the date DTG plus 3TC-dual therapy was first started in our cohort, June 30^th^, 2015, through November 30^th^, 2020. For each outcome, we excluded participants who initiated ART with a CD4/CD8 ratio above the cutoff point. Participants were censored if they changed their initial ART regimen or at the last study visit, fixed at 48 + 12 weeks (range 48-60) from ART initiation. We defined virologic failure as two consecutive HIV RNA measurements >50 copies/mL after virologic suppression. This study was approved by the Institutional Review Board of the Carlos III Health Institute located in Madrid and by the Ethics Committee at University Hospital Ramón y Cajal (ceic.hrc@salud.madrid.org).

### Statistical Analysis

We matched each participant who started 2DR with up to four participants who received 3DR based on variables selected according to prior knowledge, including age at cohort entry (within a 5-year range), sex, transmission category, educational level, country of origin, baseline HIV RNA (<100.000 copies/mm^3^, >100.000 copies/mm^3^), AIDS diagnosis, and pre-ART nadir CD4/CD8 ratio (within a 0.10 range). Subsequently, we fitted generalized estimating equation (GEE) models to allow for clustered data analysis (participants with 2DR and their matches) to assess the risk of ratio normalization at 48 weeks for each cutoff. Covariate balance after matching was evaluated by determining the mean differences between variable values for both ART groups. Survival curves were estimated with the Kaplan–Meier method. All statistical analyses were performed using Stata v. 17.0 (StataCorp LP College Station, TX, USA).

## Results

Of the 3,318 individuals who started BIC or DTG-based ART (675 and 2,643, respectively) after June 30^th^, 2015, 326 initiated dual therapy with DTG plus 3TC. **Table 1** summarizes the characteristics of the matched individuals included in each of the three analyses for cutoffs of 0.5, 1.0, and 1.5 (485, 805, and 924 participants, respectively). The distribution of covariates was similar in the three models. Overall, the study sample was representative of a medium-aged population (median age 37 years) with a higher representation of men (88%) and a median CD4/CD8 ratio around 0.30. No participant presented virological failure during follow-up. **Appendix Table S1** shows the baseline characteristics of the individuals not included in the analyses because they were not matched, CD4/CD8 information was missing, or the CD4/CD8 ratio was above the cutoff point at baseline.

**Table 1:** Population baseline characteristics according to the ART group.

	CD4/CD8 ratio >0.5		CD4/CD8 ratio >1.0		CD4/CD8 ratio >1.5	
	Dual therapy (n=108)	Triple therapy (n=377)	Dual therapy (n=176)	Triple therapy (n=629)	Dual therapy (n=198)	Triple therapy (n=726)
**Age, median (IQR)**	37 (30, 46)	36 (29, 43)	37 (30, 46)	36 (29, 44)	37 (31, 45)	37 (30, 44)
**Gender, n (%)**						
Male	92 (85)	332 (88)	153 (87)	558 (89)	174 (88)	645 (89)
Female	16 (15)	45 (12)	23 (13)	71 (11)	24 (12)	81 (11)
**Mode of transmission, n (%)**						
MSM	73 (68)	273 (72)	120 (68)	462 (73)	136 (69)	528 (73)
Heterosexual	29 (27)	89 (24)	47 (27)	149 (24)	52 (26)	174 (24)
IDU	1 (1)	1 (1)	3 (2)	6 (1)	4 (2)	11 (2)
Unknown	5 (5)	11 (3)	6 (3)	12 (2)	6 (3)	13 (2)
**Origin, n (%)**						
Spain	51 (47)	175 (46)	84 (48)	299 (48)	100 (51)	363 (50)
Western Europe	7 (6)	25 (7)	15 (9)	55 (9)	16 (8)	57 (8)
Eastern Europe	0 (0)	0 (0)	0 (0)	0 (0)	0 (0)	0 (0)
Sub-Saharan Africa	5 (5)	14 (4)	11 (6)	31 (5)	9 (5)	32 (4)
Northern Africa	2 (2)	5 (1)	3 (2)	6 (1)	3 (2)	6 (1)
Latin America	41 (38)	150 (40)	61 (35)	231 (37)	68 (34)	260 (36)
Other	2 (2)	8 (2)	2 (1)	7 (1)	2 (1)	8 (1)
**Education level, n (%)**						
No studies	2 (2)	5 (1)	5 (3)	15 (3)	5 (3)	20 (3)
Primary	17 (16)	57 (15)	22 (13)	79 (13)	22 (11)	79 (11)
Secondary	19 (18)	70 (19)	31 (18)	109 (17)	37 (19)	136 (19)
High school	29 (27)	102 (27)	47 (27)	169 (27)	52 (27)	189 (26)
University	24 (22)	89 (24)	42 (24)	158 (25)	49 (25)	186 (26)
Other	3 (3)	7 (2)	5 (3)	12 (3)	6 (3)	18 (2)
Unknown	14 (13)	47 (13)	24 (14)	83 (13)	27 (14)	98 (14)
**AIDS diagnosis, n (%)**	19 (18)	56 (15)	28 (16)	82 (13)	27 (14)	88 (12)
**Nadir CD4/CD8 ratio, median (IQR)**	0.26 (0.16, 0.39)	0.27 (0.16, 0.40)	0.32 (0.19, 0.50)	0.33 (0.19, 0.50)	0.35 (0.20, 0.53)	0.35 (0.21, 0.53)
**Nadir CD4+ cell count (cells/μL), median (IQR)**	268 (138, 444)	293 (151, 456)	311 (151, 459)	307 (178, 465)	337 (185, 483)	326 (196, 471)
**CD8+ cell count zenith (cells/μL), median (IQR)**	1338 (1041, 1709)	1332 (1023, 1725)	1263 (950, 1636)	1277 (946, 1650)	1248 (949, 1642)	1235 (902, 1666)
**HIV RNA >100.000 copies/μL, n (%)**	59 (55)	214 (57)	87 (49)	302 (48)	94 (47)	344 (47)
**Maximum HIV RNA (copies/μL), median (IQR)**	117604 (24180, 391820)	120976 (31000, 371535)	99250 (30085, 363000)	93900 (22860, 288000)	91924 (26900, 335000)	90400 (25918, 320000)
**Virologic failure during follow-up, n (%)**	0 (0)	0 (0)	0 (0)	0 (0)	0 (0)	0 (0)
**INSTI**						
Dolutegravir	–	300 (80)	–	508 (81)	–	562 (77)
Bictegravir	–	77 (20)	–	121 (19)	–	164 (23)
**NRTI backbone**						
ABC + 3TC	–	200 (53)	–	346 (55)	–	397 (55)
TDF + FTC	–	82 (22)	–	130 (21)	–	131 (18)
TAF + FTC	–	95 (25)	–	153 (24)	–	198 (27)

Participants matched according to age at cohort entry (within a 5-year range), sex, transmission category, educational level, country of origin, baseline HIV RNA (<100.000 copies/mm3, >100.000 copies/mm3), AIDS diagnosis, and pre-ART nadir CD4/CD8 ratio (within a 0.10 range). For each outcome, we excluded participants who initiated ART with a CD4/CD8 ratio above the cutoff point. All comparisons within the same cutoff point yielded p-values >0.05.

3TC, lamivudine; ABC, abacavir; ART, antiretroviral therapy; FTC, emtricitabine; IDU, injecting drug use; INSTI, integrase strand transfer inhibitor; MSM, men who have sex with men; NRTI, nucleoside reverse transriptase inhibitor; TAF, tenofovir alafenamide; TDF, tenofovir disoproxil fumarate.

**Figure 1:**
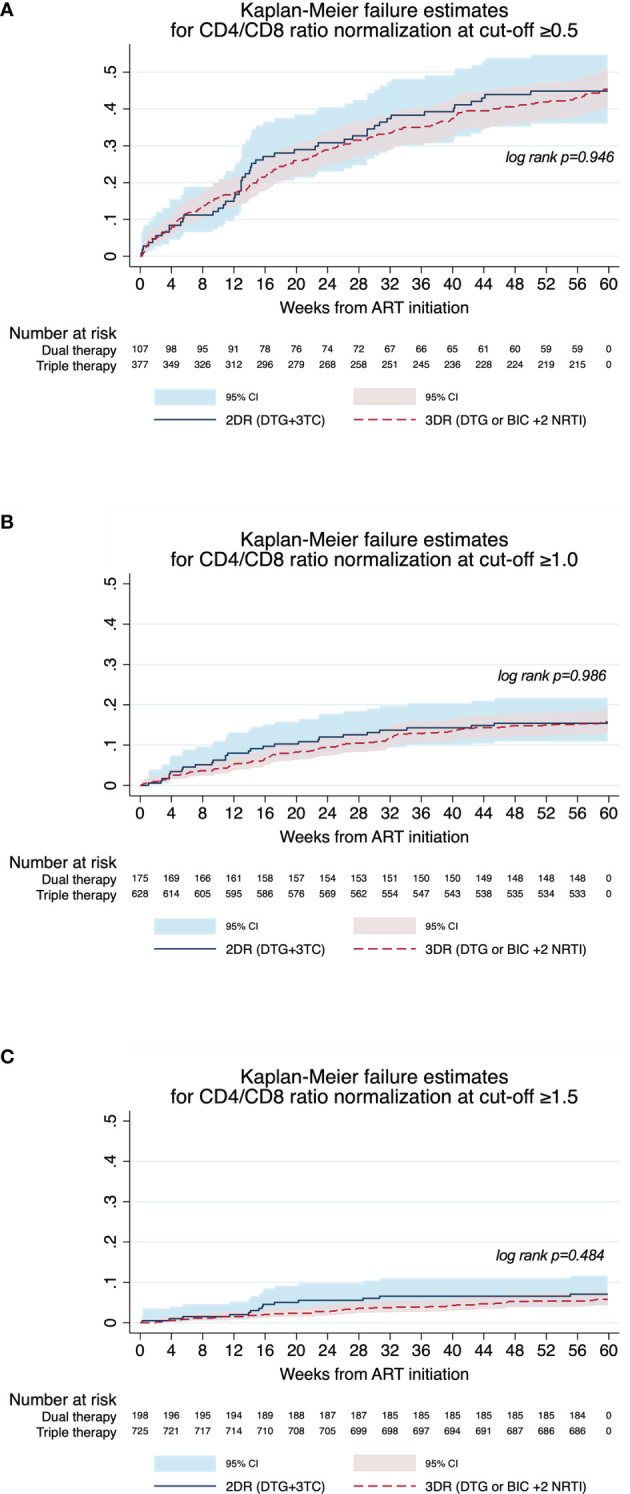
Kaplan-Meier survival estimates for CD4/CD8 ratio normalization at 0.5, 1, and 1.5 cutoffs in participants with dual and triple ART. The graph shows survival estimates for CD4/CD8 normalization for the 0.5 **(A)**, 1.0 **(B)** and 1.5 **(C)** cutoff points, during the 48 + 12 weeks of follow-up. The p-values were obtained using the log-rank test.

At 48 weeks, 45% of participants achieved a CD4/CD8 ratio >0.5, 15% achieved a ratio >1.0, and 6% achieved a ratio >1.5 (**Figure 1**). After matching according to the covariates of interest, GEE models yielded a similar risk of reaching a CD4/CD8 ratio >0.5 (OR 1.00, 95% CI 0.67 - 1.50), CD4/CD8 >1.0 (OR 1.03, 95% CI 0.68 - 1.58), and CD4/CD8 >1.5 (OR 0.86, 95% CI 0.48 - 1.54) between both treatment strategies (**Appendix Table S2**). **Figure 1** shows Kaplan-Meier survival plots of estimated overall CD4/CD8 normalization at cut-offs of 0.5, 1.0, and 1.5. There were no differences between 2DR and 3DR in the incidence ratio of CD4/CD8 ratio normalization at 0.5, 1.0 and 1.5 cut-offs (**Appendix Table S3**).

## Discussion

In this observational study of a prospective cohort of ART-naïve PLHIV, we found similar CD4/CD8 ratio recovery rates at 48 weeks between 2DR with DTG plus 3TC and 3DR based on DTG or BIC, measured at three different cutoff points (0.5, 1 and 1.5).

Persistently low CD4/CD8 ratios during ART have been strongly associated with morbidity and mortality in PLHIV (2). Therefore, given that the design of clinical trials evaluating the development of non-AIDS comorbidities according to the ART strategy is unlikely, evaluation of the effect of therapies on surrogate markers, such as the CD4/CD8 ratio, is justified.

The current interest in the study of the CD4/CD8 ratio is reflected in the different works that evaluate the evolution of this marker according to baseline predictors (12–14), and the impact achieved on this outcome with both pharmacological (15) and non-pharmacological strategies (16). Although data are showing a greater benefit in terms of CD4/CD8 ratio recovery after ART initiation with INSTI-based regimens, there are still little data on the different impact of starting 2DR vs. 3DR on this biomarker, and the vast majority have focused on the switch setting (8–11). Given the recent approval of the combination of DTG plus 3TC in the naïve patient, there is no data yet on the impact of this strategy on the recovery of this marker. Studies that have evaluated the effects of simplifying from 3DR to 2DR on the CD4/CD8 ratio included different antiretroviral classes and have yielded mixed results. While some studies have found no differences in the evolution of this marker after changing from 3DR to 2DR (8), others have reported both a possible worsening (9) and an improvement (10) in the CD4/CD8 ratio after simplification.

Previous work on this cohort showed a greater effect of INSTI-based 3DR initiation on the CD4/CD8 ratio during the first year, with significant differences in the trajectories of ratio recovery between different ART groups (7). However, because inflammation is slow to change in virologically suppressed individuals (17), further analyses will be needed to assess the impact of these different therapeutic strategies on CD4/CD8 ratio recovery in the longer term (18).

Given the large sample size and prospective nature of the CoRIS cohort, we were able to select matched participants according to the main determinants of ART choice. In addition, we mitigated the potential bias of different follow-up periods among participants by including participants on modern antiretroviral regimens, limited from the start of 2DR use in the second semester of 2015.

As in any observational study, residual confounding may have affected our study due to differences between dual and triple ART initiators. Therefore, we performed a rigorous matching of factors that could confound the causal effect of both treatment strategies on CD4/CD8 ratio normalization. After the matching process, we found a consistent pattern of similarity between groups, evidencing their exchangeability. However, we could only assess differences only after 48 weeks of ART, so next studies should evaluate potential differences that might become apparent after a longer follow-up.

This study found no differences in CD4/CD8 ratio recovery rates during the first 48 weeks after ART initiation with 2DR or 3DR INSTI-based therapies. Evaluating the effect of different therapeutic interventions on CD4/CD8 ratio changes will help decision-making as persistently low CD4/CD8 ratios despite optimal HIV-RNA suppression have been associated with a worse prognosis in PLHIV.

## Data Availability Statement

The data analyzed in this study is subject to the following licenses/restrictions: Data cannot be shared due to ethical restrictions. The data can be requested upon a substantiated request with a research protocol to Carlos III Health Research Institute. Requests to access these datasets should be directed to proyectoscoris@isciii.es.

## Ethics Statement

The studies involving human participants were reviewed and approved by Institutional Review Board of the Carlos III Health Institute and Ethics Committee at University Hospital Ramón y Cajal (ceic.hrc@salud.madrid.org). The patients/participants provided their written informed consent to participate in this study.

## Author Contributions

JM-S: Conceptualization-Equal, Data curation-Equal, Formal analysis-Equal, Investigation-Equal, Methodology-Equal, Writing – original draft-Equal; RR: Investigation-Equal, Validation-Equal, Writing – review and editing-Equal; EM: Investigation-Equal, Writing – review and editing-Equal; MS-C: Investigation-Equal, Writing – review and editing-Equal; AMu: Formal analysis-Equal, Methodology-Equal, Writing – review and editing-Equal; LL-C: Investigation-Equal, Writing – review and editing-Equal; JB: Investigation-Equal, Writing – review and editing-Equal; JP: Investigation-Equal, Writing – review and editing-Equal; ÁMe: Methodology-Equal, Writing – review and editing-Equal; SC: Investigation-Equal, Writing – review and editing-Equal; SM: Investigation-Equal, Supervision-Equal, Validation-Equal, Writing – review and editing-Equal; SS-V: Conceptualization-Equal, Investigation-Equal, Methodology-Equal, Project administration-Equal, Supervision-Equal, Validation-Equal, Writing – review and editing-Equal. All authors contributed to the article and approved the submitted version.

## Funding

This work was supported by the Spanish AIDS Research Network (RIS) through the Red Temática de Investigación Cooperativa en Sida (RD06/006, RD12/0017/0018 and RD16/0002/0006), Instituto de Salud Carlos III projects AC17/00019, PI18/00154, COV20/00349, and ICI20/00058, and co-financed by ISCIII- Subdirección General de Evaluación and by Fondo Europeo de Desarrollo Regional (FEDER). The funders had no role in the study design, data analysis, or in the interpretation of the results.

## Conflict of Interest

JM-S reports personal fees from ViiV Healthcare, Janssen Cilag, Gilead Sciences, and MSD and non-financial support from ViiV Healthcare, Jannsen Cilag, and Gilead Sciences, outside the submitted work. SS-V reports personal fees from ViiV Healthcare, Janssen Cilag, Gilead Sciences, and MSD as well as non-financial support from ViiV Healthcare and Gilead Sciences and research grants from MSD and Gilead Sciences, outside the submitted work. SM reports grants, personal fees and non-financial support from ViiV Healthcare, personal fees and non-financial support from Janssen, grants, personal fees and non-financial support from MSD, grants, personal fees and non-financial support from Gilead, outside the submitted work.

MS-C reports personal fees and non-financial support from Gilead Sciences and MSD, outside the submitted work. JRB has carried out consulting work for AbbVie, Bristol Myers Squibb, Gilead Sciences, Janssen, Merck and ViiV Healthcare and has received compensation for lectures from AbbVie, Bristol Myers Squibb, Gilead Sciences, Janssen, Merck and ViiV Healthcare, as well as grants and payments for the development of educational presentations for Gilead Sciences, Bristol Myers Squibb and ViiV Healthcare.

The remaining authors declare that the research was conducted in the absence of any commercial or financial relationships that could be construed as a potential conflict of interest.

## Publisher’s Note

All claims expressed in this article are solely those of the authors and do not necessarily represent those of their affiliated organizations, or those of the publisher, the editors and the reviewers. Any product that may be evaluated in this article, or claim that may be made by its manufacturer, is not guaranteed or endorsed by the publisher.

## References

[1] Cahn P Madero JS Arribas JR Antinori A Ortiz R Clarke AE. Dolutegravir Plus Lamivudine Versus Dolutegravir Plus Tenofovir Disoproxil Fumarate and Emtricitabine in Antiretroviral-Naive Adults With HIV-1 Infection (GEMINI-1 and GEMINI-2): Week 48 Results From Two Multicentre, Double-Blind, Randomised, non-Inferior. Lancet (2019) 393:143–55. doi: 10.1016/S0140-6736(18)32462-0 30420123

[2] Serrano-Villar S Sainz T Lee SA Hunt PW Sinclair E Shacklett BL. HIV-Infected Individuals With Low CD4/CD8 Ratio Despite Effective Antiretroviral Therapy Exhibit Altered T Cell Subsets, Heightened CD8+ T Cell Activation, and Increased Risk of Non-AIDS Morbidity and Mortality. PloS Pathog (2014) 10:e1004078. doi: 10.1371/journal.ppat.1004078 24831517 PMC4022662

[3] Mussini C Lorenzini P Cozzi-Lepri A Lapadula G Marchetti G Nicastri E. CD4/CD8 Ratio Normalisation and non-AIDS-Related Events in Individuals With HIV Who Achieve Viral Load Suppression With Antiretroviral Therapy: An Observational Cohort Study. Lancet HIV (2015) 2:98–106. doi: 10.1016/S2352-3018(15)00006-5 26424550

[4] Castilho JL Shepherd BE Koethe J Turner M Bebawy S Logan J. CD4+/CD8+ Ratio, Age, and Risk of Serious Noncommunicable Diseases in HIV-Infected Adults on Antiretroviral Therapy. AIDS (2016) 30:899–908. doi: 10.1097/QAD.0000000000001005 26959354 PMC4785819

[5] European AIDS Clinical Society (EACS) Guidelines (2021). Available at: http://www.eacsociety.org (Accessed 14 Feb 2022). v11.0, October 2021.

[6] Panel on Antiretroviral Guidelines for Adults and Adolescents. Guidelines for the Use of Antiretroviral Agents in Adults and Adolescents With HIV. Department of Health and Human Services. Available at: https://clinicalinfo.hiv.gov/sites/default/files/guidelines/documents/AdultandAdolescentGL.pdf (Accessed 28 Feb 2022). Available at: http://hivinfo.nih.gov. (Accessed 28 February 2022).

[7] Serrano-Villar S Martínez-Sanz J Ron R Talavera-Rodríguez A Fernández-Felix BM Herrera S. Effects of First-Line Antiretroviral Therapy on the CD4/CD8 Ratio and CD8 Cell Counts in CoRIS: A Prospective Multicentre Cohort Study. Lancet HIV (2020) 7:e565–73. doi: 10.1016/S2352-3018(20)30202-2 32763219

[8] Capetti AF Orofino G Paladini L Sterrantino G DiGiambenedetto S De Socio GV. Does Simplification to Dolutegravir-Based Dual Regimens Impact on the CD4+/CD8+ T-Cell Ratio? AIDS (2018) 32:1083–4. doi: 10.1097/QAD.0000000000001784 29698323

[9] Mussini C Lorenzini P Cozzi-Lepri A Marchetti G Rusconi S Gori A. Switching to Dual/Monotherapy Determines an Increase in CD8+ in HIV-Infected Individuals: An Observational Cohort Study. BMC Med (2018) 16:79. doi: 10.1186/s12916-018-1046-2 29807541 PMC5972434

[10] Monsalvo M Vallejo A Fontecha M Vivancos MJ Vizcarra P Casado JL. CD4/CD8 Ratio Improvement in HIV-1-Infected Patients Receiving Dual Antiretroviral Treatment. Int J STD AIDS (2019) 30:656–62. doi: 10.1177/0956462419834129 30961467

[11] Figueroa MI Camino-Zuñiga A Belaunzaran-Zamudio PF Sierra Madero J Andrade Villanueva J Arribas JR. The Effect of Protease Inhibitor-Based Dual Antiretroviral Regimens on CD4/CD8 Ratio During the First Year of Therapy in ART-Naïve Patients With HIV-Infection. HIV Med (2021) 22:254–61. doi: 10.1111/hiv.13008 33336523

[12] Novak RM Armon C Battalora L Buchacz K Li J Ward D. HIV Outpatient Study (HOPS) Investigators. Aging, Trends in CD4/CD8 Ratio and Clinical Outcomes With Persistent HIV Suppression in the HIV Outpatient Study (HOPS). AIDS (2022). doi: 10.1097/QAD.0000000000003171 PMC11004734 35013081

[13] Ngcobo S Molatlhegi RP Osman F Ngcapu S Samsunder N Garrett NJ. Pre-Infection Plasma Cytokines and Chemokines as Predictors of HIV Disease Progression. Sci Rep (2022) 12:2437. doi: 10.1038/s41598-022-06532-w 35165387 PMC8844050

[14] Li B Zhang L Liu Y Xiao J Li C Fan L. A Novel Prediction Model to Evaluate the Probability of CD4/CD8 Ratio Restoration in HIV-Infected Individuals. AIDS (2022). doi: 10.1097/QAD.0000000000003167 35013083

[15] Lazzaro A Cacciola EG Borrazzo C Innocenti G Pietro Calvaria EN. Switching to a Bictegravir Single Tablet Regimen in Elderly People Living With HIV-1: Data Analysis From the BICTEL Cohort. Diagnostics (Basel) (2021) 12:76. doi: 10.3390/diagnostics12010076 35054243 PMC8774414

[16] Bernal E Martinez M Campillo JA Puche G Baguena C Tomás C. Moderate to Intense Physical Activity Is Associated With Improved Clinical, CD4/CD8 Ratio, and Immune Activation Status in HIV-Infected Patients on ART. Open Forum Infect Dis (2021) 9:ofab654. doi: 10.1093/ofid/ofab654 35146043 PMC8826224

[17] Wada NI Jacobson LP Margolick JB Breen EC Macatangay B Penugonda S. The Effect of HAART-Induced HIV Suppression on Circulating Markers of Inflammation and Immune Activation. AIDS (2015) 29:463–71. doi: 10.1097/QAD.0000000000000545 PMC4311407 25630041

[18] Serrano-Villar S López Huertas MR Jiménez D. Effects of Switch From 3DR to 2DR on Inflammatory Biomarkers. Available at: http://www.croiwebcasts.org/p/2021croi/croi/527 (Accessed 13 October 2021).

