# Supplementary material for: Similar CD4/CD8 Ratio Recovery After Initiation of Dolutegravir Plus Lamivudine Versus Dolutegravir or Bictegravir-Based Three-Drug Regimens in Naive Adults With HIV
Source: Front Immunol. 2022 Mar 31;13:873408. doi: 10.3389/fimmu.2022.873408 (PMC9009371; doi:10.3389/fimmu.2022.873408)
Supplement: Supplementary file 1 [file Table_1.docx]

| **Appendix**  **Table S1. Baseline characteristics of population not included in the analyses** | | | | |  |  |
| --- | --- | --- | --- | --- | --- | --- |
|  |  |  |  |  |  |  |
|  | **CD4/CD8 ratio >0.5** | | **CD4/CD8 ratio >1.0** | | **CD4/CD8 ratio >1.5** | |
|  | **Dual therapy (n=218)** | **Triple therapy (n=2660)** | **Dual therapy (n=150)** | **Triple therapy (n=2408)** | **Dual therapy (n=128)** | **Triple therapy (n=2311)** |
| **Age, median (IQR)** | 38 (32, 48) | 39 (31, 48) | 39 (32, 49) | 39 (32, 48) | 39 (32, 50) | 39 (31, 48) |
| **Gender, n (%)** |  |  |  |  |  |  |
| Male | 189 (87) | 2295 (86) | 128 (85) | 2069 (86) | 107 (84) | 1982 (86) |
| Female | 29 (13) | 365 (14) | 22 (15) | 339 (14) | 21 (16) | 329 (14) |
| **Mode of transmission, n (%)** |  |  |  |  |  |  |
| MSM | 137 (63) | 1656 (62) | 90 (60) | 1467 (61) | 74 (58) | 1401 (61) |
| Heterosexual | 68 (31) | 713 (27) | 50 (33) | 653 (27) | 45 (35) | 628 (27) |
| IDU | 7 (3) | 141 (5) | 5 (3) | 139 (6) | 4 (3) | 134 (6) |
| Unknown | 6 (3) | 150 (6) | 5 (3) | 149 (6) | 5 (4) | 148 (6) |
| **Origin, n (%)** |  |  |  |  |  |  |
| Spain | 119 (55) | 1536 (58) | 86 (57) | 1390 (58) | 70 (55) | 1335 (58) |
| Western Europe | 13 (6) | 209 (8) | 5 (3) | 191 (8) | 4 (3) | 175 (7) |
| Eastern Europe | 1 (0) | 58 (2) | 1 (1) | 51 (2) | 1 (1) | 46 (2) |
| Sub-Saharan Africa | 16 (7) | 153 (6) | 10 (7) | 144 (6) | 12 (9) | 135 (6) |
| Northern Africa | 4 (2) | 29 (1) | 3 (2) | 27 (1) | 3 (2) | 23 (1) |
| Latin America | 62 (28) | 643 (24) | 42 (28) | 580 (24) | 35 (27) | 569 (25) |
| Other | 3 (1) | 32 (1) | 3 (2) | 25 (1) | 3 (2) | 28 (1) |
| **Education level, n (%)** |  |  |  |  |  |  |
| No studies | 10 (5) | 75 (3) | 7 (5) | 72 (3) | 7 (5) | 71 (3) |
| Primary | 10 (5) | 251 (9) | 5 (3) | 229 (9) | 5 (4) | 213 (9) |
| Secondary | 31 (14) | 379 (14) | 19 (13) | 343 (14) | 13 (10) | 326 (14) |
| High school | 69 (32) | 765 (29) | 51 (34) | 686 (29) | 46 (36) | 674 (29) |
| University | 60 (28) | 669 (26) | 42 (28) | 612 (25) | 35 (27) | 582 (25) |
| Other | 6 (3) | 39 (1) | 4 (3) | 32 (1) | 3 (2) | 36 (2) |
| Unknown | 32 (15) | 482 (18) | 22 (15) | 434 (18) | 19 (15) | 409 (18) |
| **AIDS diagnosis, n (%)** | 30 (13) | 347 (13) | 21 (14) | 321 (13) | 22 (17) | 315 (14) |
| **Nadir CD4/CD8 ratio, median (IQR)** | 0.44 (0.25, 0.68) | 0.39 (0.21, 0.61) | 0.48 (0.22, 0.83) | 0.38 (0.20, 0.61) | 0.44 (0.19, 0.77) | 0.37 (0.20, 0.61) |
| **Nadir CD4+ cell count (cells/μL), median (IQR)** | 380 (225, 504) | 331 (195, 476) | 392 (230, 542) | 329 (192, 476) | 347 (168, 500) | 324 (188, 473) |
| **Acme CD8+ cell count (cells/μL), median (IQR)** | 1107 (720, 1559) | 1182 (856, 1634) | 1088 (663, 1559) | 1176 (852, 1640) | 1085 (672, 1478) | 1192 (854, 1637) |
| **HIV RNA >100.000 copies/μL, n (%)** | 93 (43) | 1199 (45) | 65 (43) | 1111 (46) | 58 (45) | 1069 (46) |
| **Maximum HIV RNA (copies/μL), median (IQR)** | 74091 (26000, 256000) | 81808 (22761, 277800) | 81316 (20826, 238000) | 85543 (24500, 290000) | 84020 (21870, 256000) | 84880 (23600, 281000) |
| **Virologic failure during follow-up, n (%)** | 0 (0) | 0 (0) | 0 (0) | 0 (0) | 0 (0) | 0 (0) |
| **INSTI** |  |  |  |  |  |  |
| Dolutegravir | - | 2062 (78) | - | 1854 (77) | - | 1800 (78) |
| Bictegravir | - | 598 (22) | - | 554 (23) | - | 511 (22) |
| **NRTI backbone** |  |  |  |  |  |  |
| ABC + 3TC | - | 1368 (51) | - | 1222 (51) | - | 1171 (51) |
| TDF + FTC | - | 568 (21) | - | 520 (22) | - | 519 (22) |
| TAF + FTC | - | 724 (27) | - | 666 (28) | - | 621 (27) |
| Individuals not included in the analyses because they were not matched, CD4/CD8 information was missing, or the CD4/CD8 ratio was above the cutoff point at baseline.All comparisons within the same cutoff point yielded p-values >0.05. | | | | | | |
| Abbreviations: 3TC, lamivudine; ABC, abacavir; ART, antiretroviral therapy; FTC, emtricitabine; IDU, injecting drug use; INSTI, integrase strand transfer inhibitor; MSM, men who have sex with men; NRTI, nucleoside reverse transriptase inhibitor; TAF, tenofovir alafenamide; TDF, tenofovir disoproxil fumarate. | | | | | | |

| **Table S2. Generalized estimating equation (GEE) models for ratio normalization at 48 weeks** | | | |
| --- | --- | --- | --- |
| **ART group** | **OR** | **95% CI** | **p** |
| **CD4/CD8 ratio > 0.5** |  |  |  |
| Dual therapy | Ref. | - | - |
| Triple therapy | 1.00 | 0.67, 1.50 | 0.985 |
| **CD4/CD8 ratio > 1.0** |  |  |  |
| Dual therapy | Ref. | - | - |
| Triple therapy | 1.03 | 0.68, 1.58 | 0.871 |
| **CD4/CD8 ratio > 1.5** |  |  |  |
| Dual therapy | Ref. | - | - |
| Triple therapy | 0.86 | 0.48, 1.54 | 0.612 |
| Participants matched according to age at cohort entry (within a 5-year range), sex, transmission category, educational level, country of origin, baseline HIV RNA (<100.000 copies/mm3, >100.000 copies/mm3), AIDS diagnosis, and pre-ART nadir CD4/CD8 ratio (within a 0.10 range). | | | |
| Abbreviations: CI, confidence interval; OR, odds ratio; Ref, reference category. | | | |

| **Table S3. Incidence rates of CD4/CD8 normalization at different cutoff points** | | |
| --- | --- | --- |
|  | **Incidence rate (per 1000 person-months)** | **95% CI** |
| **CD4/CD8 ratio > 0.5** |  |  |
| Dual therapy | 44 | 33, 58 |
| Triple therapy | 43 | 37, 50 |
| **CD4/CD8 ratio > 1.0** |  |  |
| Dual therapy | 12 | 8, 17 |
| Triple therapy | 12 | 10, 14 |
| **CD4/CD8 ratio > 1.5** |  |  |
| Dual therapy | 5 | 3, 8 |
| Triple therapy | 4 | 3, 5 |
| Abbreviations: CI, confidence interval | | |

**CENTERS AND RESEARCHERS INVOLVED IN CoRIS**

**Executive committee**

Santiago Moreno, Inma Jarrín, David Dalmau, Maria Luisa Navarro, María Isabel González, Federico García, Eva Poveda, José Antonio Iribarren, Félix Gutiérrez, Rafael Rubio, Francesc Vidal, Juan Berenguer, Juan González, M Ángeles Muñoz-Fernández.

**Fieldwork data management and analysis**

Inmaculada Jarrín, Belén Alejos, Cristina Moreno, Carlos Iniesta, Luis Miguel García Sousa, Nieves Sanz Pérez, Marta Rava.

**BioBanK HIV Hospital General Universitario Gregorio Marañón**

M Ángeles Muñoz-Fernández, Irene Consuegra Fernández.

**Hospital General Universitario de Alicante (Alicante)**

Esperanza Merino, Gema García, Irene Portilla, Iván Agea, Joaquín Portilla, José Sánchez-Payá, Juan Carlos Rodríguez, Lina Gimeno, Livia Giner, Marcos Díez, Melissa Carreres, Sergio Reus, Vicente Boix, Diego Torrús.

**Hospital Universitario de Canarias (San Cristóbal de la Laguna)**

Ana López Lirola, Dácil García, Felicitas Díaz-Flores, Juan Luis Gómez, María del Mar Alonso, Ricardo Pelazas, Jehovana Hernández, María Remedios Alemán, María Inmaculada Hernández.

**Hospital Universitario Central de Asturias (Oviedo)**

Víctor Asensi, Eulalia Valle, María Eugenia Rivas Carmenado, Tomás Suárez-Zarracina Secades, Laura Pérez Is.

**Hospital Universitario 12 de Octubre (Madrid)**

Rafael Rubio, Federico Pulido, Otilia Bisbal, Asunción Hernando, Lourdes Domínguez, David Rial Crestelo, Laura Bermejo, Mireia Santacreu.

**Hospital Universitario de Donostia (Donostia-San Sebastián)**

José Antonio Iribarren, Julio Arrizabalaga, María José Aramburu, Xabier Camino, Francisco Rodríguez-Arrondo, Miguel Ángel von Wichmann, Lidia Pascual Tomé, Miguel Ángel Goenaga, Mª Jesús Bustinduy, Harkaitz Azkune, Maialen Ibarguren, Aitziber Lizardi, Xabier Kortajarena, Mª Pilar Carmona Oyaga, Maitane Umerez Igartua.

**Hospital General Universitario De Elche (Elche)**

Félix Gutiérrez, Mar Masiá, Sergio Padilla, Catalina Robledano, Joan Gregori Colomé, Araceli Adsuar, Rafael Pascual, Marta Fernández, José Alberto García, Xavier Barber, Vanessa Agullo Re, Javier García Abellán, Reyes Pascual Pérez, María Roca.

**Hospital Universitari Germans Trias i Pujol (Can Ruti) (Badalona)**

Roberto Muga, Arantza Sanvisens, Daniel Fuster.

**Hospital General Universitario Gregorio Marañón (Madrid)**

Juan Berenguer, Juan Carlos López Bernaldo de Quirós, Isabel Gutiérrez, Margarita Ramírez, Belén Padilla, Paloma Gijón, Teresa Aldamiz-Echevarría, Francisco Tejerina, Francisco José Parras, Pascual Balsalobre, Cristina Diez, Leire Pérez Latorre, Chiara Fanciulli.

**Hospital Universitari de Tarragona Joan XXIII (Tarragona)**

Francesc Vidal, Joaquín Peraire, Consuelo Viladés, Sergio Veloso, Montserrat Vargas, Montserrat Olona, Anna Rull, Esther Rodríguez-Gallego, Verónica Alba, Alfonso Javier Castellanos, Miguel López-Dupla.

**Hospital Universitario y Politécnico de La Fe (Valencia)**

Marta Montero Alonso, José López Aldeguer, Marino Blanes Juliá, María Tasias Pitarch, Iván Castro Hernández, Eva Calabuig Muñoz, Sandra Cuéllar Tovar, Miguel Salavert Lletí, Juan Fernández Navarro.

**Hospital Universitario La Paz/IdiPAZ**

Juan González-García, Francisco Arnalich, José Ramón Arribas, José Ignacio Bernardino de la Serna, Juan Miguel Castro, Ana Delgado Hierro, Luis Escosa, Pedro Herranz, Víctor Hontañón, Silvia García-Bujalance, Milagros García López-Hortelano, Alicia González-Baeza, María Luz Martín-Carbonero, Mario Mayoral, María José Mellado, Rafael Esteban Micán, Rocío Montejano, María Luisa Montes, Victoria Moreno, Ignacio Pérez-Valero, Guadalupe Rúa Cebrián, Berta Rodés, Talia Sainz, Elena Sendagorta, Natalia Stella Alcáriz, Eulalia Valencia.

**Hospital San Pedro Centro de Investigación Biomédica de La Rioja (CIBIR) (Logroño)**

José Ramón Blanco, José Antonio Oteo, Valvanera Ibarra, Luis Metola, Mercedes Sanz, Laura Pérez-Martínez.

**Hospital Universitario Miguel Servet (Zaragoza)**

Piedad Arazo, Gloria Sampériz.

**Hospital Universitari MutuaTerrassa (Terrasa)**

David Dalmau, Angels Jaén, Montse Sanmartí, Mireia Cairó, Javier Martinez-Lacasa, Pablo Velli, Roser Font, Marina Martínez, Francesco Aiello.

**Complejo Hospitalario de Navarra (Pamplona)**

Maria Rivero Marcotegui, Jesús Repáraz, María Gracia Ruiz de Alda, María Teresa de León Cano, Beatriz Pierola Ruíz de Galarreta.

**Corporació Sanitària Parc Taulí (Sabadell)**

María José Amengual, Gemma Navarro, Manel Cervantes Garcia, Sonia Calzado Isbert, Marta Navarro Vilasaro.

**Hospital Universitario de La Princesa (Madrid)**

Ignacio de los Santos, Jesús Sanz Sanz, Ana Salas Aparicio, Cristina Sarria Cepeda, Lucio García-Fraile Fraile, Enrique Martín Gayo.

**Hospital Universitario Ramón y Cajal (Madrid)**

Santiago Moreno, José Luis Casado Osorio, Fernando Dronda Núñez, Ana Moreno Zamora, Maria Jesús Pérez Elías, Carolina Gutiérrez, Nadia Madrid, Santos del Campo Terrón, Sergio Serrano Villar, María Jesús Vivancos Gallego, Javier Martínez Sanz, Usua Anxa Urroz, Tamara Velasco.

**Hospital General Universitario Reina Sofía (Murcia)**

Enrique Bernal, Alfredo Cano Sanchez, Antonia Alcaraz García, Joaquín Bravo Urbieta, Ángeles Muñoz Perez, María Jose Alcaraz, María del Carmen Villalba.

**Hospital Nuevo San Cecilio (Granada)**

Federico García, José Hernández Quero, Leopoldo Muñoz Medina, Marta Álvarez, Natalia Chueca, David Vinuesa García, Clara Martínez-Montes, Carlos Guerrero Beltrán, Adolfo de Salazar González, Ana Fuentes López.

**Centro Sanitario Sandoval (Madrid)**

Jorge Del Romero, Montserrat Raposo Utrilla, Carmen Rodríguez, Teresa Puerta, Juan Carlos Carrió, Mar Vera, Juan Ballesteros, Oskar Ayerdi.

**Hospital Clínico Universitario de Santiago (Santiago de Compostela)**

Antonio Antela, Elena Losada.

**Hospital Universitario Son Espases (Palma de Mallorca)**

Melchor Riera, María Peñaranda, Mª Angels Ribas, Antoni A Campins, Carmen Vidal, Francisco Fanjul, Javier Murillas, Francisco Homar, Helem H Vilchez, Maria Luisa Martin, Antoni Payeras.

**Hospital Universitario Virgen de la Victoria (Málaga)**

Jesús Santos, Cristina Gómez Ayerbe, Isabel Viciana, Rosario Palacios, Carmen Pérez López, Carmen Maria Gonzalez-Domenec.

**Hospital Universitario Virgen del Rocío (Sevilla)**

Pompeyo Viciana, Nuria Espinosa, Luis Fernando López-Cortés.

**Hospital Universitario de Bellvitge (Hospitalet de Llobregat)**

Daniel Podzamczer, Arkaitz Imaz, Juan Tiraboschi, Ana Silva, María Saumoy, Paula Prieto.

**Hospital Universitario Valle de Hebrón (Barcelona)**

Esteban Ribera, Adrián Currán.

**Hospital Costa del Sol (Marbella)**

Julián Olalla Sierra, Javier Pérez Stachowski, Alfonso del Arco, Javier de la torre, José Luis Prada, José María García de Lomas Guerrero.

**Hospital General Universitario Santa Lucía (Cartagena)**

Onofre Juan Martínez, Francisco Jesús Vera, Lorena Martínez, Josefina García, Begoña Alcaraz, Amaya Jimeno.

**Complejo Hospitalario Universitario a Coruña (CHUAC) (A Coruña)**

Ángeles Castro Iglesias, Berta Pernas Souto, Álvaro Mena de Cea.

**Hospital Universitario Basurto (Bilbao)**

Josefa Muñoz, Miren Zuriñe Zubero, Josu Mirena Baraia-Etxaburu, Sofía Ibarra Ugarte, Oscar Luis Ferrero Beneitez, Josefina López de Munain, Mª Mar Cámara López, Mireia de la Peña, Miriam Lopez, Iñigo Lopez Azkarreta.

**Hospital Universitario Virgen de la Arrixaca (El Palmar)**

Carlos Galera, Helena Albendin, Aurora Pérez, Asunción Iborra, Antonio Moreno, María Angustias Merlos, Asunción Vidal, Marisa Meca.

**Hospital de la Marina Baixa (La Vila Joiosa)**

Concha Amador, Francisco Pasquau, Javier Ena, Concha Benito, Vicenta Fenoll, Concepción Gil Anguita, José Tomás Algado Rabasa.

**Hospital Universitario Infanta Sofía (San Sebastián de los Reyes)**

Inés Suárez-García, Eduardo Malmierca, Patricia González-Ruano, Dolores Martín Rodrigo, Mª Pilar Ruíz Seco.

**Hospital Universitario de Jaén (Jaén)**

Mohamed Omar Mohamed-Balghata, María Amparo Gómez Vidal.

**Hospital San Agustín (Avilés)**

Miguel Alberto de Zarraga.

**Hospital Clínico San Carlos (Madrid)**

Vicente Estrada Pérez, Maria Jesús Téllez Molina, Jorge Vergas García, Juncal Pérez-Somarriba Moreno.

**Hospital Universitario Fundación Jiménez Díaz (Madrid)**

Miguel Górgolas, Alfonso Cabello, Beatriz Álvarez, Laura Prieto.

**Hospital Universitario Príncipe de Asturias (Alcalá de Henares)**

José Sanz Moreno, Alberto Arranz Caso, Cristina Hernández Gutiérrez, María Novella Mena.

**Hospital Clínico Universitario de Valencia (València)**

María José Galindo Puerto, Ramón Fernando Vilalta, Ana Ferrer Ribera.

**Hospital Reina Sofía (Córdoba)**

Antonio Rivero Román, Antonio Rivero Juárez, Pedro López López, Isabel Machuca Sánchez, Mario Frias Casas, Ángela Camacho Espejo.

**Hospital Universitario Severo Ochoa (Leganés)**

Miguel Cervero Jiménez, Rafael Torres Perea.

**Nuestra Señora de Valme (Sevilla)**

Juan A Pineda, Pilar Rincón Mayo, Juan Macías Sánchez, Nicolás Merchanté Gutierrez, Luis Miguel Real, Anais Corma Gomez, Marta Fernández Fuertes, Alejandro González-Serna.

**Hospital Álvaro Cunqueiro (Vigo)**

Eva Poveda, Alexandre Pérez, Manuel Crespo, Luis Morano, Celia Miralles, Antonio Ocampo, Guillermo Pousada.
